# Brazilian multicenter study for the evaluation of patients’ satisfaction of blood glucose self-monitoring with BGStar^®^ blood glucose meter in insulinized patients with diabetes mellitus type 1 and 2

**DOI:** 10.1186/s13098-016-0180-2

**Published:** 2016-09-13

**Authors:** Miguel Nasser Hissa

**Affiliations:** Centro de Pesquisas em Diabetes e Doenças Endócrino-metabólicas, Medical School UNICHRISTUS, Rua Canuto de Aguiar, 500/100, Meireles, Fortaleza, Ceará 60160120 Brazil

**Keywords:** Blood glucose meter, Self-monitoring of blood glucose, Diabetes, Insulinized patients, BGStar

## Abstract

**Background:**

Diabetes mellitus (DM) is considered a global epidemic, and patient self-management education and support are critical in preventing and reducing the risk of complications. Self-monitoring of blood glucose (SMBG) is essential for care of individuals with DM, helping patients to achieve and maintain target blood glucose levels. The purpose of this study is to compare the satisfaction of insulinized DM patients on SMBG with use of investigational blood glucose meter (BGM) versus their routine device.

**Methods:**

A national, multicenter, open-label, phase 4 study was conducted on patients with type 1 or 2 DM under insulin therapy regimen, who were asked to use investigational BGM instead of their usual BGM device. The study was performed in 12 centers in Brazil for 12 weeks, with an extension period of 12 weeks. The primary endpoint was to measure the variation on the patients’ level of satisfaction with investigational versus routine BGM, between visits, using a Visual Analogue Scale (VAS). Secondary endpoints addressed handling aspects, satisfaction, adherence and level of functionality and safety of investigational BGM.

**Results:**

The study included 292 patients (36.6 % DM1 and 63.4 % DM2), mean age 50.9 years old (±17.3 years), 57.5 % females. There was statistically significant improvement in global satisfaction with investigational BGM compared with routine BGM according to VAS [mean VAS score raised from 78.8 mm (SD = 18.0) to 90.8 mm (SD = 12.2) between visits]. After 12 weeks, level of satisfaction with investigational BGM according to questionnaires was superior to routine BGM regardless of age group (p < 0.001), type of DM (p < 0.001) or insulin regimen (p < 0.001). Investigational BGM was also regarded as safe, with 10 patients (3.4 %) reporting a total of 13 adverse events during the study.

**Conclusions:**

Levels of satisfaction during SMBG were higher with use of investigational BGM and the device was deemed safe and easy to handle.

## Background

Diabetes mellitus (DM) is considered as a global epidemic, with a worldwide prevalence estimated in 9 % among adults aged >18 years in 2014 [1] and 1.5 million deaths reported in 2012 as a direct cause of the disease [2]. This is a complex, chronic illness and both types (DM1 and DM2) require continuous medical care with multifactorial risk-reduction strategies beyond glycemic control.

Patient self-management education and support are critical to preventing and reducing the risk of long-term complications. Among several actions that should be taken to manage DM is self-monitoring of blood glucose (SMBG). SMBG is an essential tool for the care of individuals with the disease, being important to help patients achieve and maintain target blood glucose levels in order to reduce the risk of diabetes-related complications [3].

SMBG is recognized as a fundamental component of effective diabetic self-management [4–6]. In patients with type 1 diabetes (DM1) it was associated with better results in controlling blood glucose levels [7]. In patients with type 2 diabetes (DM2) in use of insulin therapy, it optimizes blood glucose control [8, 9]. Nevertheless, benefits of SMBG in patients with DM2 who are not using insulin are still controversial. Current evidence on this subject is mixed, with studies showing both significant and insignificant glycemic benefits resulting from SMBG [8, 10, 11].

In any case, frequent SMBG is important to adequately manage type 1 or 2 DM. When used properly, glucose meters for self-measurement of blood glucose allow diabetes patients to determine their blood glucose level and can guide insulin dose adjustment [3, 11]. The Diabetes Control and Complications Trial among people with type 1 DM clearly showed the efficacy of tight glycemic control in reducing diabetic complications [12, 13].

Modern glucose meters are small, easy to handle, and require very little blood volume (usually less than 5 μl), providing accuracy at rates of 95 %. Overall performance of SMBG systems is a combination of the analytical performance of the instrument, proficiency of the patient, and quality of the test strips and the underlying measurement technology, thereby allowing patients and clinicians to monitor glycemic control [3].

The objective of this study was to compare the patients’ satisfaction with the blood glucose meter (BGM) used in daily routine for self-monitoring blood glucose versus an investigational BGM device.

## Methods

A national, multicenter, open-label, one arm, phase 4 study was conducted on patients with type 1 or 2 DM under insulin therapy regimen. The study was performed in 12 centers in Brazil from April 2013 to December 2014, and compared patient’s satisfaction with the blood glucose meter BGStar^®^ (Sanofi-Aventis, Frankfurt, Germany) after 12 weeks of use in comparison to the previous BGM used in their daily routine, for SMBG. At the end of this period, patients could opt to continue using the investigational BGM for an additional period of 12 weeks (extension period). As the study design is a single arm, there was no need for randomization.

The study comprised five visits and five questionnaires to be completed by patients, according to the study visit (Appendix). A questionnaire based on a Visual Analogue Scale (VAS) was used to assess patients’ global satisfaction with BGM, and the others were seven-item questionnaires that evaluated patient general preferences on handling features of BGMs (Questionnaire 1, answered at visit 1); on handling features of previous BGM (Questionnaire 2, answered at visit 1); on handling features of investigational BGM after 12 and 24 weeks of use (Questionnaire 3, answered at visits 4 and 5); and adherence to the use of investigational BGM and its adequate operation along the study (Questionnaire 4, answered at visits 2–5).

The study selected patients with ≥18 years old, both male and female, with type 1 or type 2 diabetes, receiving insulin for at least 2 years; with a stable insulin regimen and in use of any BGM for at least 6 months. The study also required that the patient had the ability to perform blood glucose self-monitoring. Patients on CSII were excluded from the study.

The primary endpoint was the comparison of the patients’ satisfaction with the investigational BGM after 12 weeks of use versus their satisfaction with the previous BGM used in daily routine. Satisfaction was based on questionnaires completed by patients, such as Visual Analogue Scale (VAS) and preferences on handling features of BGM (speed to obtain results, size, appearance, reliability and insulin dose adjustments). Secondary endpoints included comparison of patients’ global satisfaction with the investigational BGM after 24 weeks of use compared to baseline and to week 12 levels of satisfaction. Also, it included the evaluation of: patients’ general preferences on handling features of the investigational BGM and of previous BGM; patient general preferences on handling features of the investigational BGM after 12 and 24 weeks of use; adherence to the use of the investigational BGM and its adequate operation along the study; frequency of hypoglycemic episodes during the study (including extension period) related to the study procedures; number of Product Technical Complaints (PTCs) during the study, including extension period; and safety information.

This study was performed in compliance with Good Clinical Practices, including the archiving of essential documents, and with the ICH Harmonized Tripartite Guideline on the Structure and Content of Clinical Study Reports, dated July 1996, using QSOP-004712 Version 3.0. The study was approved by all local Institutional Review Boards (IRBs; by each participant site). As this study was not conducted in the USA, and following the sponsor’s policy, the study was not disclosed on ClinicalTrials.gov.

### Statistical methods

All patients who signed the informed consent form (ICF) were considered as screened. Patient who signed the ICF and were not a screen failure were included in intent-to-treat (ITT) population. All ITT patients who had used at least once the investigational BGM were also considered in the modified intent-to-treat (m-ITT) population. Safety population consisted of all enrolled patients. All efficacy analyses were based on m-ITT population, and all safety analyses on ITT population.

Quantitative data were summarized by descriptive statistics such as mean, standard deviation, minimum and maximum and the categorical data were summarized by absolute (n) and relative (%) frequency of occurrence.

All results were based on total number of patients with available information (i.e. patients who attended the visit and for whom the specific data was not missing). The type I error adopted for the statistical tests was 5 %. All analyses were conducted using SAS version 9.4.

Satisfaction analysis (overall satisfaction with the investigational BGM) were assessed using the Visual Analogue Scale (VAS) and a specific questionnaire of satisfaction with seven items, applied at initial visit (V_1_), final visit after 3 months (V_4_) and extension visit after 6 months (V_5_). For each efficacy endpoint (VAS and the sum of the seven-item satisfaction questionnaire), a model of analysis of variance (ANOVA) was applied with a fixed factor of repeated measures (VISIT), since the same patient is evaluated in two or more visits. The main objective of the ANOVA model was to identify if there was a “VISIT effect”, i.e. to identify if there was any change in the patients satisfaction across V_1_, V_4_ and V_5_.

Global satisfaction was also analyzed in subgroups (age, type of diabetes and insulin delivery system). An ANOVA model was also applied in this case, including the subgroup factor and the interaction with VISIT. The objective was to identify if a possible change observed along the visits in the patients’ satisfaction was similar by each subgroup category. When the factor VISIT was significant in an ANOVA model including the extension period, multiple comparison tests were carried out.

Each of the individual seven-item questionnaire scores were also analyzed in order to see any specific change from V_1_ (satisfaction compared with previous BGM) and V_4_ (satisfaction after 12 weeks using the investigational BGM), through the non-parametric Wilcoxon rank sum test.

## Results

### Population

**Fig. 1:**
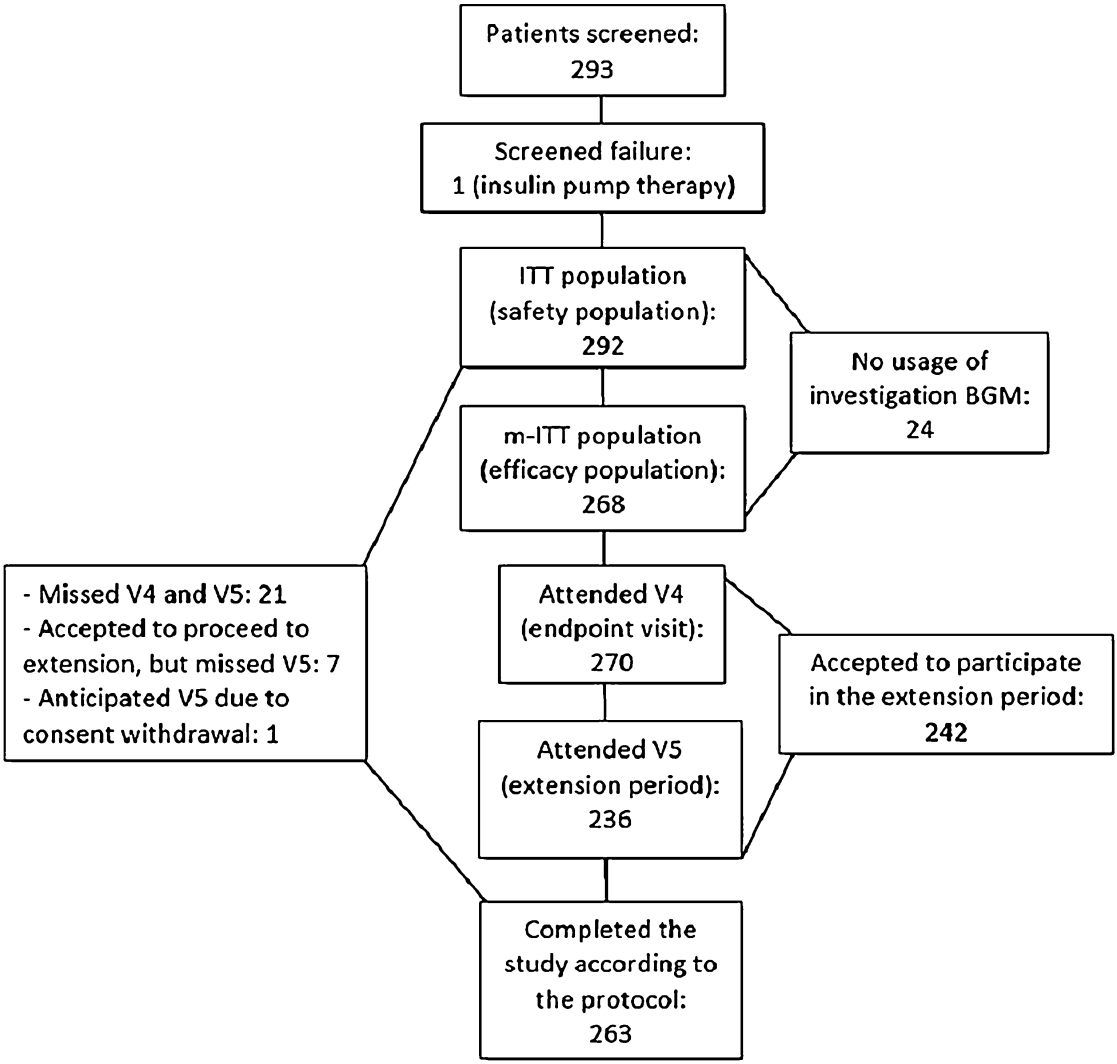
Patient flow diagram. *ITT* intention-to-treat; *m*-*ITT* modified intention-to-treat; *V4* visit 4; *V5* visit 5; *BGM* blood glucose meter

Between 5th April 2013 and 16th June 2014, 293 patients were enrolled in the study. All of them had signed the informed consent form, but one was excluded (screen failure) due to the use of insulin pump therapy. ITT population was similar to the safety population and comprised 292 patients. From this total, 268 patients (91.8 %) constituted the modified-ITT (m-ITT) population, as 8.2 % of the patients did not use at least once the investigational BGM (Fig. 1).

From the ITT population, 270 (92.5 %) patients attended the endpoint visit (V_4_), 82.9 % accepted to participate in the extension period and 80.8 % indeed attended V_5_. Twenty-nine (9.9 %) patients did not complete the study according to the protocol, wherein seven of them had agreed to proceed to extension visit, but did not perform V_5_ (Fig. 1). Reasons for the patients did not complete the study were: lost to follow up (n = 16), consent withdrawal (n = 12) and death (n = 1).

Four patients were considered as protocol deviation. Two of them did not fulfill the criterion “type 2, receiving insulin for at least 2 years with a stable insulin regimen for at least 6 months”, one patient did not fulfill the criterion “use of any BGM for at least 6 months, with ability to perform blood glucose self-monitoring” and the other one did not fulfill the criterion “age ≥18 years old”.

Age of patients ranged from 17.9 to 86.1 years, with mean age of 50.9 (SD = 17.3) and median of 54.7 years. Despite the protocol inclusion criteria “age ≥18 years old”, one patient aging 17.9 years was screened and performed all planned visits; this patient was considered for both ITT and m-ITT analyses. Regarding gender, 124 (42.5 %) patients were male and 168 (57.5 %) female. Body mass index (BMI) ranged between 17.6 and 47.7 kg/m^2^, with mean of 28.9 kg/m^2^ (SD = 5.3). Means of systolic and diastolic pressure were 129.9 (SD = 20.5) and 78.4 (SD = 12.4) mmHg, respectively (Table 1).

**Table 1:** Demographic and clinical characteristics of patients (ITT)

Characteristics		Total of available patients (n)
*Age (years)*		
Mean ± SD	50.9 ± 17.3	
Minimum–maximum	17.9–86.1	
N of patients (%)		
<25 years	27 (9.2 %)	
≥25 to <65 years	197 (67.5 %)	
≥65 years	68 (23.3 %)	292
*Sex*—*n (%)*		
Male	124 (42.5 %)	
Female	168 (57.5 %)	292
*BMI (kg/m* ^*2*^ *)*		
Mean ± SD	28.9 ± 5.3	
Minimum–maximum	17.6–47.7	289
*Systolic BP (mmHg)*		
Mean ± SD	129.9 ± 20.5	
Minimum–maximum	90–240	291
*Diastolic BP*		
Mean ± SD	78.4 ± 12.4	
Minimum–maximum	51–170	291
*Type of diabetes mellitus*—*n (%)*		
Type 1	107 (36.6 %)	
Type 2	185 (63.4 %)	292
*Insulin regimen application* ^a^—*n (%)*		
Basal-bolus	177 (60.6 %)	
Others	115 (39.4 %)	292

^a^At baseline visit

*ITT* intent-to-treat; *BMI* body mass index

Most patients (185; 63.4 %) had type 2 diabetes mellitus. Mean time of diabetes diagnosis was 15.0 years (SD = 9.1). Basal-bolus was the insulin regimen application at baseline for most patients (60.6 %) (Table 1).

### Patients’ satisfaction results

Adherence to investigational BGM was evaluated at visits (V_2_, V_4_ and V_5_) by an investigator. Adherence was categorized, according to the protocol definition, as Total (BGM was used at least once a day), Partial (BGM was used at least once a day, between 2 and 6 days of the week) and No Adherence (BGM was used at most once a week). Most of patients (71.3 %) totally adhered to BGM at V_2_, but this number decreased at V_4_ (62.8 %) and extension visit V_5_ (52.8 %), while Partial adherence remained stable (Table 2).

**Table 2:** Adherence to BGM according to the study center, by visit (ITT)

Visit	Study period		Extension period
	V2	V4	V5
Week	Week 4	Week 12	Week 24
*Adherence*			
Total	194 (71.3 %)	167 (62.8 %)	122 (52.8 %)
Partial	61 (22.4 %)	67 (25.2 %)	56 (24.2 %)
No	17 (6.3 %)	32 (12.0 %)	53 (22.9 %)
Total of available patients	272 (100 %)	266 (100 %)	231 (100 %)

*Total* BGM was used at least once a day; *Partial* BGM was used at least once a day, between 2 and 6 days of the week; *No* BGM was used at most once a week

The global satisfaction with the investigational BGM after 12 weeks of use was compared to the global satisfaction with previous BGM, based on VAS score (0–100 mm) and on the sum of a seven-item questionnaire. In the satisfaction questionnaire based on VAS, higher values on the scale are associated to higher satisfaction; moreover, in the analysis of the seven-items questionnaire, a lower score is associated to higher satisfaction. From the 268 patients of m-ITT population, 258 patients attended V_1_ and V_4_, and answered the global satisfaction questionnaire. Of these, 176 (68.2 %) assigned a higher VAS score after 12 weeks of use of investigational BGM compared with previous BGM, 47 (18.2 %) indicated no change and 35 (13.6 %) assigned a lower score after 12 weeks. A statistically significant improvement of satisfaction was observed when using investigational BGM for 12 weeks: mean VAS score raised from 78.8 mm (SD = 18.0) at V_1_ to 90.8 mm (SD = 12.2) at V_4_. Mean difference on VAS score was 11.9 (SD = 20.4; p < 0.001; 95 % CI 9.4–14.4 mm) (Table 3).

**Table 3:** Overall satisfaction with previous BGM and investigational BGM through Visual Analogue Scale (VAS), during the study period (m-ITT)

Visit	Study period		Difference
	V1	V4	
Day/week	Day 1	Week 12	VAS_V4_–VAS_V1_
*VAS (0*–*100* *mm)*			
Mean ± SD	78.8 ± 18	90.8 ± 12.2	11.9 ± 20.4
Median	80.0	94.0	10.0
Q1–Q3	70 to 90	86 to 100	0 to 20
Minimum–maximum	0 to 100	14 to 100	−61 to 90
CI 95 %	76.6 to 81.0	89.3 to 92.2	9.4 to 14.4
Total of available patients	258 (100 %)	258 (100 %)	258 (100 %)
p value (visit)	p < 0.001		

*ITT* intention-to-treat; *SD* standard deviation; *CI* confidence interval

Mean change of VAS score from V_1_ to V_4_ was also analyzed for the subgroups: Type of diabetes, Insulin regimen application and Age (Table 4). There was no observed statistically significant effect (p = 0.221) for the interactions VISIT*TYPE, indicating that the increase in mean satisfaction with the use of investigation BGM for 12 weeks was similar for patients with T1DM (14.0 ± 20.8 mm) and T2DM (10.8 ± 20.2 mm). Regarding the subgroup insulin regimen application, interaction VISIT*REGIMEN was not statically significant (p = 0.133), showing that the increase in the satisfaction with the investigational BGM was similar for patients at basal-bolus (13.5 ± 20.0 mm) and at other application regimens (9.5 ± 20.9 mm).

**Table 4:** Overall satisfaction with previous BGM and with investigational BGM after 12 weeks of use, based on Visual Analogue Scale (VAS) by age, diabetes type and insulin regimen (m-ITT)

Study period			Total of available patients	Change from baseline	ANOVA p value
Visit	V1	V4			
Day/week	Day 1	Week 12		VAS_V4_–VAS_V1_	
	Mean ± SD	Mean ± SD		Mean ± SD	VISIT: p < 0.001
*Diabetes type*					
Type 1	73.1 ± 17.2	87.2 ± 13.4	93	14 ± 20.8	VISIT^a^TYPE: p = 0.221
Type 2	82 ± 17.7	92.8 ± 11	165	10.8 ± 20.2	
*Insulin application regimen*					
Basal-bolus	76 ± 18	89.4 ± 12	158	13.5 ± 20	VISIT: p < 0.001
Other	83.3 ± 17.2	92.8 ± 12.2	100	9.5 ± 20.9	VISIT^a^REGIMEN: p = 0.133
*Age group*					
<25 years	72 ± 19.7	89.6 ± 11.4	24	17.6 ± 19.9	VISIT: p < 0.001
25 to <65 years	77.9 ± 18.6	91.2 ± 11.4	171	13.3 ± 20.5	VISIT^a^AGE: p = 0.023
≥65 years	83.8 ± 14.2^a^	90 ± 14.4	63	6.2 ± 19.6	

^a^In the group with ≥65 years the initial evaluation was already high, thus the difference wasn’t significant

Moreover, a statistically significant effect was observed for the interaction VISIT*AGE (p = 0.023). The increase observed in the satisfaction based on VAS after 12 weeks of use of investigational BGM was inversely proportional to the age. By observing the same three age groups in the visit V_1_ (satisfaction with the previous BGM), we observed that younger patients (age <25 years) were more dissatisfied than older patients (age ≥65 years), according to Turkey’s method for multiple comparisons (17.6 ± 19.9 versus 6.2 ± 19.6 mm, respectively; global significance level = 0.05) for the age groups in V_1_. In the satisfaction evaluation performed at V_4_ (after 12 weeks of use of investigational BGM), there was no statistically significant difference among the three age groups. It is also important to note that in the group of patients with ≥65 years the initial evaluation was already high, thus the difference was not significant. Also, at week 12, all age groups showed very similar satisfaction levels according to VAS scores means, which may indicate that satisfaction with the investigational BGM may increase with its use, and the subsequent raise in the ability to handle it.

Regarding global satisfaction based on a seven-item questionnaire sum, 253 patients from the m-ITT population (n = 268) answered all questionnaires, and were therefore included in the analysis. The mean sum of questionnaire scores was 15.8 (SD = 3.8) at V_1_ and 12.0 (SD = 3.4) at V_4_, meaning an improvement of satisfaction when using investigational BGM for 12 weeks (p < 0.001). There was no interaction effect of any of the subgroups tested—VISIT*TYPE (p = 0.113), VISIT*REGIMEN (p = 0.439) and VISIT*AGE (p = 0.560)—meaning that changing of satisfaction score from V_1_ to V_4_ was similar for all age groups, both type 1 and type 2 diabetes, and also basal-bolus versus other insulin application regimens. Global satisfaction with investigational BGM after 12 weeks of use was also compared with previous BGM through each of the individual seven-item questionnaire. All seven-item scores (except item 6: “On average, how often do you stop to take the blood glucose tests recommended by your doctor with the current BGM/investigational BGM?”) decreased significantly (p < 0.001), indicating better satisfaction after 12 weeks usage of investigational BGM.

Global satisfaction levels after 24 weeks using investigational BGM was compared to those obtain with previous BGM and investigational BGM after 12 weeks of use. Levels were based on VAS score (0–100 mm) and on the sum of a seven-item questionnaire. From the 268 patients of m-ITT population, 224 patients attended V_1_, V_4_ and V_5_ and answered the global satisfaction questionnaire based on VAS; and 219 answered the seven-item questionnaire. Mean VAS score raised from 78.6 mm (SD = 18.4) at V_1_ to 91.3 mm (SD = 11.3) at V_4_ and remained almost the same at V_5_, with mean score of 90.3 mm (SD = 13.5) (Table 5). Mean sum of satisfaction questionnaire score was 15.7 (SD = 3.9) at V_1_, 11.8 (SD = 3.3) at V_4_ and 12.0 (SD = 3.6) at V_5_. Multiple comparisons showed higher mean score at V_1_ than V_5_, indicating an increase in the satisfaction after using the investigational BGM for 24 weeks, but similar mean scores at V_4_ and V_5_, showing that the satisfaction was maintained from V_4_ to V_5_ (Table 6).

**Table 5:** Overall satisfaction with previous BGM and investigational BGM based on Visual Analogue Scale (VAS), during the study period and extension (m-ITT)

Study period			Extension period
Visit	V1	V4	V5
Day/week	Day 1	Week 12	Week 24
*VAS (0*–*100* *mm)*			
Mean ± SD	78.6 ± 18.4	91.3 ± 11.3	90.3 ± 13.5
Median	80.0	94.5	95.0
Q1–Q3	70–90.5	90–100	86–100
Minimum–maximum	0–100	14–100	10–100
Total of available patients	224 (100 %)	224 (100 %)	224 (100 %)
*p* value (visit)	p < 0.001

**Table 6:** Sum of scores of satisfaction questionnaire with previous BGM and investigational BGM, during the study period and extension (m-ITT)

Study period			Extension period
Visit	V1	V4	V5
Day/week	Day 1	Week 12	Week 24
*Sum of scores*			
Mean ± SD	15.7 ± 3.9	11.8 ± 3.3	12 ± 3.6
Median	16.0	11.0	11.0
Q1–Q3	13–18	9–14	9–14
Minimum–maximum	7–29	7–24	7–26
Total of available patients	219 (100 %)	219 (100 %)	219 (100 %)
p-value (visit)	p < 0.001		

### Safety Results

Ten (3.4 %) patients reported a total of 13 adverse events during the study period, including the 12-week extension. One patient reported four events, and all the others presented only one event each. One (0.3 %) patient had an adverse event considered serious: fall with mild traumatic head injury due to symptoms of hypoglycemia; this event was the only one related to the investigational BGM (product technical complaint, PTC). This case was analyzed by the legal manufacturer; the malfunction of the device was detected and subsequently corrected. Two (0.7 %) patients reported adverse events related to hypoglycemia: 1 (0.3 %) patient at V_5_ (considered serious, with blood glucose level ≤36 mg/dl), and 1 (0.3 %) patient at V_2_ (considered non-serious, with blood glucose level ≤70 mg/dl). None of the events resulted in permanent discontinuation of BGM use (Table 7).

**Table 7:** Adverse events (ITT)

	N. of patients (%)	N. of events
N. (%) of patients with any AE	10 (3.4 %)	13
SAE	1 (0.3 %)	1
*Criterion for SAE*		
Life-threatening	1 (0.3 %)	1
*Intensity*		
Mild	8 (2.7 %)	11
Moderate	1 (0.3 %)	1
Severe	1 (0.3 %)	1
*Treatment/corrective therapy*		
No	8 (2.7 %)	11
Yes	2 (0.7 %)	2
*Related with investigation BGM technical complaint*		
Yes (serious)	1 (0.3 %)	1
N. of patients without any AE	282 (96.6 %)	
Total of available patients	292 (100 %)	

*AE* adverse event; *SAE* serious adverse eventa

There were 21 (7.2 %) patients with at least one PTC, resulting in 27 reported PTCs. Nineteen PTCs were reported by the patients, 4 by the investigators and four by the study staff.

## Discussion

Self-management of diabetes remains the cornerstone of diabetes care. An overall self-management strategy would be greatly beneficial to people with diabetes as well as to their families. Major clinical trials of insulin-treated patients have included SMBG as part of the multifactorial interventions to demonstrate the benefit of intensive glycemic control on diabetes complications. SMBG is thus an integral component of effective therapy. Also, evidence points out that more frequent self-monitoring of blood glucose levels is associated with clinically and statistically better glycemic control regardless of diabetes type or therapy [14].

SMBG should be considered and evaluated in conjunction with all other aspects of diabetes self-management and care. The Canadian Diabetes Association (CDA) clinical practice guidelines for prevention and management of diabetes recommend that SMBG should be individualized for each person with diabetes based on their circumstances and needs, and the American Diabetes Association (ADA) guidelines recommend that patient’s specific needs and goals should dictate SMBG frequency and timing [4, 15]. This would help individuals to avoid hypoglycemic episodes during dose modifications, to correctly adjust medications, to evaluate individual response to therapy and assess if glycemic targets are being achieved [4, 8, 15].

Determination of blood glucose values through BGM should be performed regularly by insulin-treated diabetic patients, and the values obtained from these glucose readings should be used to direct and guide insulin dosing on daily basis. In order to allow good treatment efficacy and safety, BGMs need to be robust, easy to use, accurate and reliable [3].

Programmatic, structured SMBG contributes to significant improvement in glycemic control in diabetic patients under insulin regimen [10]. But SMBG accuracy depends on the instrument and user, so these are important aspects to be considered. Optimal use of SMBG requires proper review and interpretation of data, both by patients and physicians: patients should be taught how to use SMBG data to adjust food intake, exercise, or pharmacological therapy to achieve specific goals as well as to monitor and prevent asymptomatic hypoglycemia and hyperglycemia [4].

In an accuracy study, conducted in accordance with the ISO 15197 standards, BGStar demonstrated reliable results compared with other glucometers available on the European market. Results of this study suggest that patients preferred choosing BGStar than other glucometers due to the facility regarding the use, storage and analysis memory available in the software [3].

In the Precision trial, BGStar demonstrated similar accuracy compared with other monitoring technologies used, with high intra-method and inter-method results, suggesting that BGStar is a precise and accurate method of glucose monitoring with many resources, including electrochemistry dynamics, which improve diabetes management experience for patients [16].

SMBG can be considered the primary technique to assess the effectiveness of the patient glycemic control plan and the satisfaction with a BGM should be a key point in the management of diabetes, particularly among insulin-treated patients. A new BGM device could potentially contribute to stimulate the patients to better control glycemia. This multicentric study conducted in Brazilian patients diagnosed with type 1 and type 2 diabetes demonstrated that the investigational BGM received positive reviews by patients regarding its speed to obtain results, size and appearance, reliability, insulin dose adjustments and an overall satisfaction score.

The limitations of this study, is that when a new intervention is introduced there will be an expected level of satisfaction because this is something new and different to what the consumer is used to. Others studies are necessary to confirm our data.

## Conclusions

The values obtained from glucose readings are used to direct and guide insulin dosing on a daily basis. Thus, frequent self-monitoring of blood glucose levels is associated with better glycemic control. In this regard, appropriate use of structured SMBG significantly improves glycemic control and facilitates more timely/aggressive treatment for patients with diabetes mellitus [10, 14].

The study showed that levels of satisfaction during SMBG were higher with the use of investigational BGM than with routine BGM and the device was deemed safe and easy to handle.

## Abbreviations

ADA: American Diabetes Association
BGM: blood glucose meter
CDA: Canadian Diabetes Association
CI: confidence interval
DM: diabetes mellitus
DM1: type 1 diabetes mellitus
DM2: type 2 diabetes mellitus
ICF: informed consent form
ITT: intent-to-treat
m-ITT: modified intent-to-treat
SD: standard deviation
SMBG: self-monitoring of blood glucose
VAS: Visual Analogue Scale
